# Crystal Structure Analysis of the Polysialic Acid Specific O-Acetyltransferase NeuO

**DOI:** 10.1371/journal.pone.0017403

**Published:** 2011-03-01

**Authors:** Eike C. Schulz, Anne K. Bergfeld, Ralf Ficner, Martina Mühlenhoff

**Affiliations:** 1 Abteilung für Molekulare Strukturbiologie, Institut für Mikrobiologie und Genetik, Georg-August-Universität Göttingen, Göttingen, Germany; 2 Institut für Zelluläre Chemie, Medizinische Hochschule Hannover, Hannover, Germany; University of Cambridge, United Kingdom

## Abstract

The major virulence factor of the neuroinvasive pathogen *Escherichia coli* K1 is the K1 capsule composed of α2,8-linked polysialic acid (polySia). K1 strains harboring the CUS-3 prophage modify their capsular polysaccharide by phase-variable O-acetlyation, a step that is associated with increased virulence. Here we present the crystal structure of the prophage-encoded polysialate O-acetyltransferase NeuO. The homotrimeric enzyme belongs to the left-handed β-helix (LβH) family of acyltransferases and is characterized by an unusual funnel-shaped outline. Comparison with other members of the LβH family allowed the identification of active site residues and proposal of a catalytic mechanism and highlighted structural characteristics of polySia specific O-acetyltransferases. As a unique feature of NeuO, the enzymatic activity linearly increases with the length of the N-terminal poly-ψ-domain which is composed of a variable number of tandem copies of an RLKTQDS heptad. Since the poly-ψ-domain was not resolved in the crystal structure it is assumed to be unfolded in the apo-enyzme.

## Introduction

Extra-intestinal pathogenic *Escherichia coli* (ExPEC) strains of the capsular serotype K1 are the predominant cause of neonatal sepsis and meningitis [1], [2]. The major virulence factor of *Escherichia coli* K1 (*E. coli* K1) is the K1 capsule which is essential for serum resistance and vital passage through the blood brain barrier [1], [3]. The capsular polysaccharide (cps) consists of α2,8-linked polysialic acid (polySia), a polyanionic homopolymer composed of up to 200 residues of the sialic acid 5-*N*-acetyl neuraminic acid (Neu5Ac) [4]. Structurally identical polySia is found in the host organism, where it occurs as a functionally important glycan modification of the neural cell adhesion molecule, NCAM, involved in brain development and synaptic plasticity [5], [6]. Thus, *E. coli* K1 disguises itself with a host sugar polymer which limits immunodetection and raises concerns against polySia-based vaccines [7], [8]. The same mechanism of molecular mimicry is used by *Neisseria meningitidis* (*N. meningitidis* or meningococcus) serogroup B, an important cause of sepsis and meningitis after the neonatal period. However, whereas α2,8-linked polySia of both group B menigococci and the human host consists exclusively of Neu5Ac residues, the polySia capsule of many *E. coli* K1 strains can be modified by O-acetylation of the Neu5Ac residues at positions O_7_ and O_9_ [9]–[11]. Although O-acetylation increases immunogenicity of the K1 capsule it was correlated with increased virulence rates in bacteraemic patients [12]. Moreover, O-acetylation impairs polySia degradation by neuraminidases and increases desiccation resistance, which may favour colonization of the intestinal tract and survival outside the host organism, respectively [11], [13].

O-acetylation of the *E. coli* K1 capsule is catalyzed by the acetyl-CoA dependent sialate O-acetyltransferase NeuO, an enzyme that is highly specific for α2,8-linked polySia with a minimum chain length of 14 residues [10], [14]. The corresponding gene is not part of the cps gene cluster but is associated with the P22-like prophage CUS-3 [9], [15]. As a result, only K1 strains harbouring CUS-3 are able to O-acetylate their capsule and the presence of CUS-3/*neuO* is tightly linked to K1 strains of the phylogenetic sequence type complex 95 comprising human invasive isolates [13].

Notably, K1 capsule O-acetylation (OAc) undergoes high-frequency phase-variation [11]. This stochastic switch between ‘on’ (OAc^+^) and ‘off’ (OAc^−^) forms is mediated by a variable number tandem repeat (VNTR) within the 5′-coding region of *neuO* [9]. The VNTR region, denoted poly-ψ-motif, is inserted two nucleotides downstream of the start codon and consists of the heptanucleotide unit 5′-AAGACTC-3′ which occurs in copy numbers between 2 and 93 [9], [13]. During replication, this region is prone to strand slippage leading to frequent changes in overall length, with gain or loss of a single tandem copy being the most frequent event [16]. Full length translation and thereby expression of active NeuO (phase ‘on’) is restricted to copy numbers that are a multiple of three, whereas all other copy numbers result in frameshifts leading to truncated, inactive protein (phase ‘off’) [9]. In the case of full length translation, every three tandem repeats are translated into an RLKTQDS heptad. Although this variable N-terminal protein extension is not a prerequisite for activity, the catalytic efficiency increases linearly with the number of RLKTQDS heptads, representing a unique mechanism for gradual regulation of enzymatic activity [9], [14].

Beyond the variable poly-ψ-domain, NeuO is characterized by the presence of imperfect tandem repeats of the hexapeptide motif [LIV]-[GAED]-X_2_-[STAV]-X, a hallmark of the hexapeptide repeat family of acyltransferases [17]. The crystal structures of several members of this family reveal that three hexapeptides fold into one coil of a left-handed parallel β-helix (LβH), which resembles a triangular prism [18], [19]. Based on this central fold, the family is also termed LβH-superfamily and comprises not only acyltransferases but also Zn-dependent γ-carbonic anhydrases [20].

O-acetylation of polySia capsules is not restricted to *E. coli* K1 but is also observed in *N. meninigitidis* serogroup C, characterized by a cps composed of α2,9-linked polySia, and serogroup W-135 and Y meningococci, encapsulated by heteropolymers composed of the disaccharide repeating unit [-6-Gal-α1,4-Neu5Ac-α2-]_n_ and [-6-Glc-α1,4-Neu5Ac-α2-}_n_], respectively [21], [22]. Whereas the capsule modifying O-acetyltransferase OatC of serogroup C belongs to the α/β-hydrolase fold family [23], serogroups W-135 and Y share an identical O-acetyltransferase termed OatWY, which belongs to the LβH-superfamily [24], [25].

In the present study, we crystallized NeuO using a protein variant with four N-terminal RLKTQDS heptads. The crystal structure shows that NeuO is a member of the LβH-family that displays an unusual quaternary structure arrangement presumably as an adaptation to accommodate the exceptionally long polySia acceptor. Comparison between the structures of NeuO and OatWY revealed highly conserved residues within the active site allowing to propose a catalytic mechanism for NeuO.

## Results

### Structure determination

**Figure 1:**
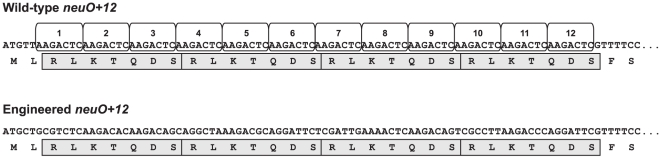
Engineering of a *neuO* gene that allows phase-stable expression of NeuO with four N-terminal heptads. Schematic representation of the poly-ψ-domain of an allelic variant encoding NeuO with four N-terminal heptads. The sequence of the naturally occurring *neuO+12* allele comprising twelve tandem copies of the sequence 5′-AAGACTC-3′ is shown in the upper panel with the nucleotide repeats shown in open boxes. RLKTQDS heptads encoded by 3 hepta-nucleotide repeats are highlighted by grey boxes. In the engineered *neuO* sequence shown in the lower panel, the 5′-region was exchanged by a sequence that still encoded four heptads but lacks any nucleotide repeats.

For structure determination of NeuO, the protein variant with four N-terminal RLKTQDS heptads was chosen. Initially, the protein was expressed from the naturally occurring allele *neuO*_+12_ which contains twelve tandem copies of the heptanucleotide unit 5′-AAGACTC-3′ encoding four RLKTQDS heptads. However, no crystals were obtained for protein expressed from this VNTR containing sequence. Since VNTRs are prone to slipped-strand DNA synthesis, *neuO* variants with altered repeat numbers can be generated when bacteria are grown for protein expression. Eventually, this may lead to trace amounts of protein with three and/or five N-terminal heptads. To omit the formation of heterogeneous protein, we engineered a *neuO* gene in which the VNTR-region was replaced by an artificial sequence stretch that still encoded four RLKTQDS heptads but lacked any nucleotide repeats (**Figure 1**). Protein expressed from this engineered sequence had a total length of 252 residues including a C-terminal His_6_-tag. Throughout the manuscript, the numbering of amino acid residues was performed with the N-terminal residue (methionine) numbered 1. Accordingly, the numbering of residues located C-terminally to the four heptads increased by 28 compared to a NeuO variant devoid of RLKTQDS heptad repeats.

Protein expressed from the engineered sequence yielded crystals that belong to space group P2_1_. The crystals contain three monomers in the asymmetric unit and diffract up to a resolution of 1.7 Å (**Table 1**). Phases were obtained by molecular replacement. The NeuO structure was refined to R-factors of 16.6% (R_free_ = 19.5%) and shows good stereochemistry (**Figure S1**). In the longest chain that could be placed into the electron density, residues 19–251 are present. By contrast, residues 1–18 encompassing the first two heptads were too flexible to be determined as indicated by increasing B-factors at all three N-termini.

**Table 1:** Data Collection and Refinement Statistics.

*Data collection*		*Refinement*	
Wavelength (Å)	0.9028	Resolution limits (Å)	37.35 – 1.7
Cell dimensions (Å)		No. of used reflections	79582
a	62.83	No. of protein atoms	5243
b	88.36	No. of ligand atoms	24
c	72.96	No of water atoms	910
α	90.0	R-factor (%)	0.1657
β	106.62	R_free_ (%)	0.1954
γ	90.0		
Space group	P2_1_	*Ramachandran plot statistics*	
Resolution range (Å)	46.01 – 1.70	Most favourable region (%)	98%
No. of reflections	327825 (46462)	Generously allowed regions (%)	2%
Average I/σ	12.4 (2.6)	Disallowed regions (%)	0%
Completeness (%)	99.8 (99.0)	*r.m.s. deviations from ideal values*	
Redundancy	3.9 (3.8)	Bond distance (Å)	0.005
R_merge_ (%)	0.064 (0.474)	Angles (°)	0.921

Values in parentheses indicate the specific values in the highest resolution shell.

R = Σ(∥F_obs_|−scale |F_model_∥)/Σ(|F_obs_|).

R_merge_ = Σ_hkl_Σ*_i_*|I*_i_*(hkl)−<I*_i_*(hkl)>|/Σ_hkl_Σ*_i_*<I*_i_*(hkl)>, where the sum *i* is over all separate measurements of the unique reflection hkl.

R_free_ as R-factor, but summed over a 4.96% test set of reflections.

### Overall structure

**Figure 2:**
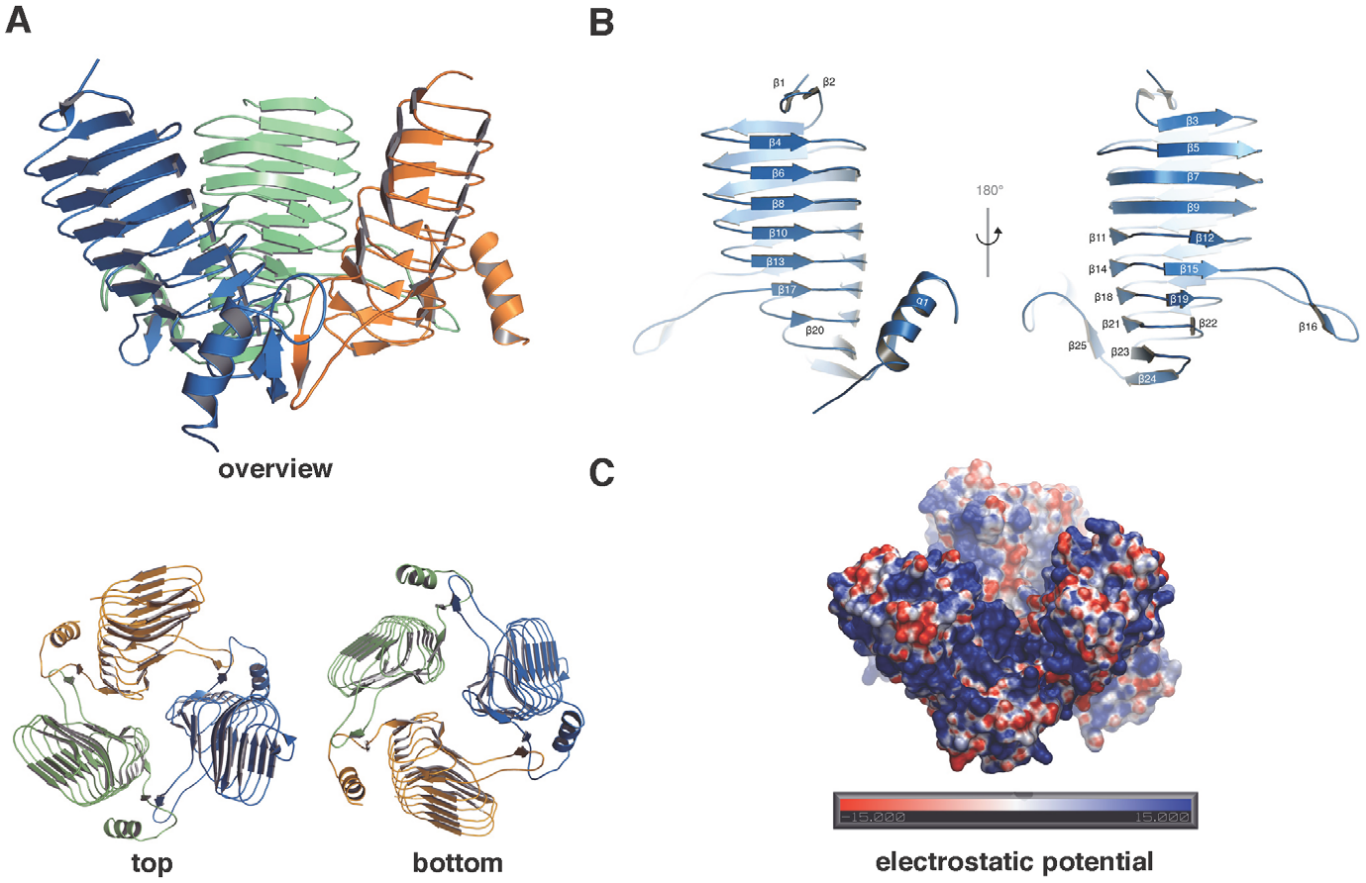
Crystal structure of NeuO. (A) Overview about the three-dimensional structure of NeuO with each monomer displayed in a different color in cartoon mode; top and bottom views are given. Monomers are tilted by almost 45° with respect to each other. (B) A single monomer of NeuO is displayed from both sides; the annotation for every secondary structure element is indicated. (C) Representation of the electrostatic surface potential of the NeuO trimer, displayed from −15 to 15 k_b_T, calculated using the program APBS. [44]. Most of the surface is positively charged (blue) suited to bind to the poly-anionic acceptor substrate polySia.

The crystal structure showed that NeuO is a homotrimeric protein with a funnel-like outline (**Figure 2A**). The overall shape is a result of the subunit arrangement characterized by an inclination of about 45° between the long axes of the monomers. While the N-termini point away from each other, giving rise to a diameter of about 65 Å, the monomers interconnect at their C-terminal section leading to a reduced diameter of about 50 Å. Each monomer forms a left-handed parallel β-helix consisting of 23 β-strands forming seven β-helical coils. At half of the length of each monomer the parallel β-helix is interrupted by a protruding loop composed of 20 residues (residues 143–163) including those forming β16. At the C-terminal end, the LβH-domain is extended by a further β-strand and a single α-helix (**Figure 2B**). Each trimerization interface is formed by the protruding loop of one subunit interacting with the bar-like ending (β25 and α1) of the adjacent subunit. Notably, intersubunit contacts are mainly formed by the anti-parallel alignment of β16 and β25.

### The LβH-domain

The predominant part of the NeuO monomer folds into an LβH-domain. However, in contrast to the canonical LβH fold, which resembles an equilateral triangular prism [18], the LβH domain of NeuO displays a more extended outline. The diameter of the parallel β-helix decreases from 27 Å at the N-terminus to about 10 Å at the C-terminus, resulting in a wedge-like shape of each monomer (**Figure 2B**). The distance between the opposing β-strands stays constantly 11.5 Å, which, in combination with the extended outline gives rise to a bean-shaped cross section of the LβH-domain (**Figure 2A**, bottom panel). Due to the inclination between the subunits, direct interactions between adjacent LβH-domains are limited to the last two β-rungs (residues 190–210), to the protruding loop and the bar-like C-terminal extension of the LβH-domain. Here the anti-parallel alignment of β-strands β16 and β25 forms hydrogen bonds between the main-chains, which closely connect the individual monomers. With the exception of a salt bridge between chain A and B (Arg174A ∶ Glu119B), which can not be detected between the other chains, intersubunit interactions mediated by the β-rungs are restricted to Asn209 contacting Gly193 and Asn209 of the adjacent chain.

### Substrate binding sites

**Figure 3:**
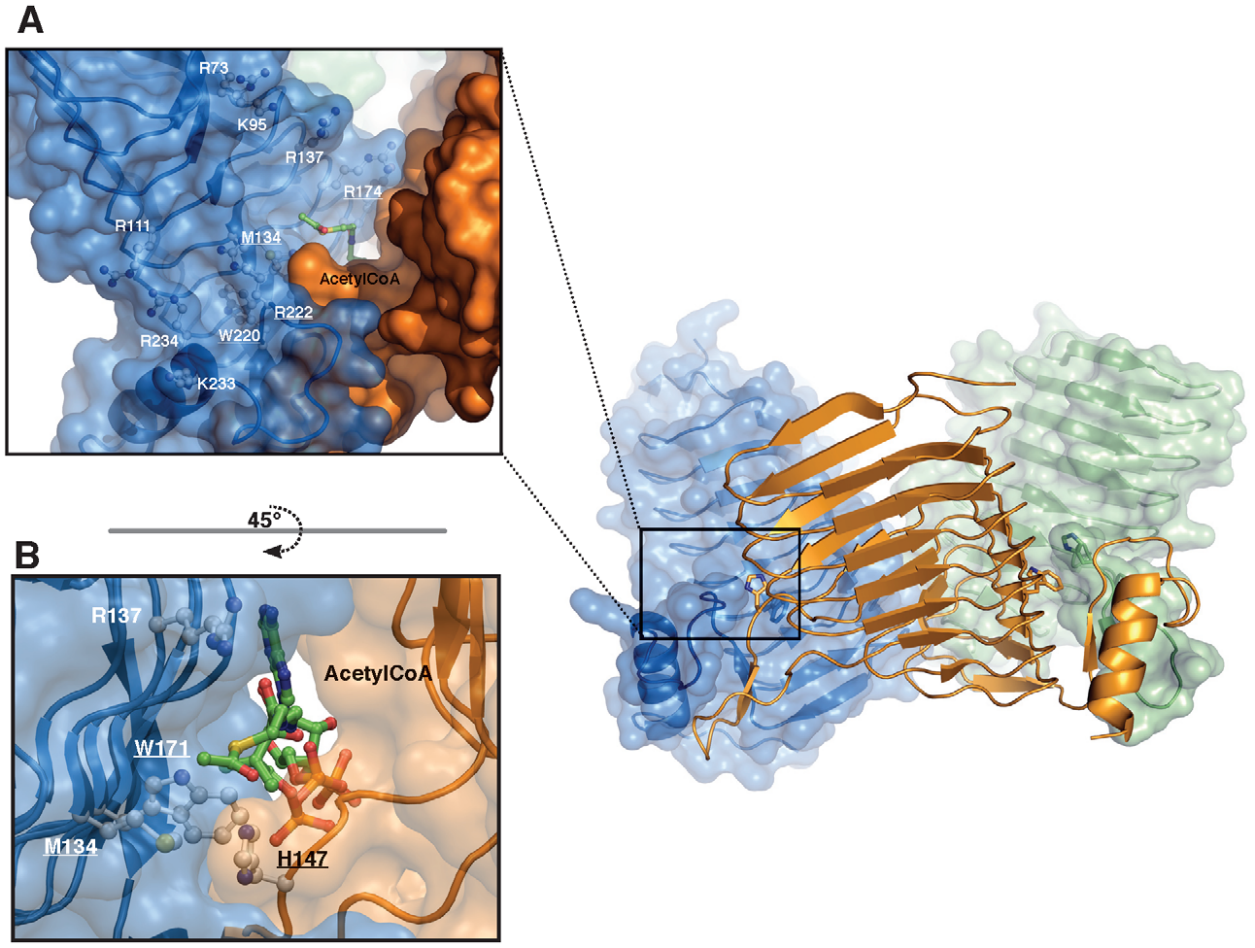
NeuO active site. (A) The active and polySia binding site of NeuO is displayed as a full molecule view with half-transparent surface for two chains (right panel). A close-up view shows the binding site in detail (left panel). Residues that are conserved between NeuO and the closely related O-acetyltransferase OatWY are underlined and shown in ball and stick mode. In addition, residues that form the proposed polySia binding site are highlighted in ball and stick mode. The donor-substrate acetyl-CoA (shown as green sticks) was modeled into the NeuO structure by superposition with the crystal structure of the acetyltransferase OatWY (PDB-ID: 2WLF) from *N. meningitidis*. (B) A rotation of 45° allows a better view into the active site located at the interface of two adjacent subunits and shows the close proximity of Met134 and Trp171 belonging to the same subunit and His147, which belongs to the adjacent monomer.

Despite close intersubunit interactions at the bottom of the trimer, a tunnel can be observed between each subunit. These tunnels, about 10 Å in diameter and about 25 Å long, pierce through the enzyme parallel to the long axis of the LβH-domain (**Figure 3**). Situated at the N-terminal outlet of each tunnel are His147 and Trp171 (corresponding to His119 and Trp143 in NeuO lacking N-terminal heptad-repeats), which have previously been reported to be catalytically important (**Figure 3B**) [14]. Structural similarity to other O-acetyltransferases of the LβH-family suggests that each tunnel forms a binding site for the donor-substrate acetyl-CoA. However, since NeuO failed to crystallize in the presence of acetyl-CoA structural evidence is missing. In order to test whether this site can harbor the donor substrate, an acetyl-CoA molecule was successfully modeled into the tunnel by superposition with the crystal structure of O-acetyltransferase OatWY of *N. meningitidis* serogroup W-135 and Y (**Figure 3**) [25]. In conclusion, presence of catalytically important residues in combination with possible donor-substrate binding, indicate that the N-terminal outlet of the observed tunnel represents the active site of NeuO.

In front of the active site, the wide inclination of the three NeuO monomers generates a platform, which is oriented perpendicular to the long axis of the donor-substrate binding tunnel (**Figure 3A**). This platform is almost exclusively positively charged (**Figure 2C**) and thus well suited to bind the polyanionic substrate polySia. Previous reports have shown that the minimum substrate length for NeuO is a Sia_14_-molecule [10], [14], suggesting an extended carbohydrate binding site. The location of positively charged residues around the active site of NeuO (Arg111, Arg234, Lys233, Arg222, Arg174, Arg137, Lys95, and Arg73) (Fig. 3A) implicates that polySia may bind along the positively charged platform, makes a sharp turn behind the active site and then further runs along the inner surface of the LβH domain towards the N-terminus of NeuO. However, co-crystallization trials with colominic acid, partially hydrolyzed polySia containing oligomers with 2 to over 60 residues, did not yield any suitable crystals, most likely due to lack of homogeneity in oligomer length. Using a defined acceptor-substrate was not feasible, since separation capacities of available chromatographic methods to isolate Sia oligomers from hydrolyzed polySia decrease with oligomer length. Accordingly, the longest commercially available substrate is currently Sia_6_ and quantity as well as homogeneity of the Sia_14_-chains separated on an anion-exchange column were not sufficient for co-crystallization experiments.

Previously, it was shown that the catalytic efficiency of NeuO increases linearly with the number of N-terminal heptads [14]. However, since the poly-ψ-domain is not visible in the electron density of the crystal structure it can be concluded that it is disordered in the apo-enzyme (**Figure S2**).

### Structural comparison between NeuO and OatWY

**Figure 4:**
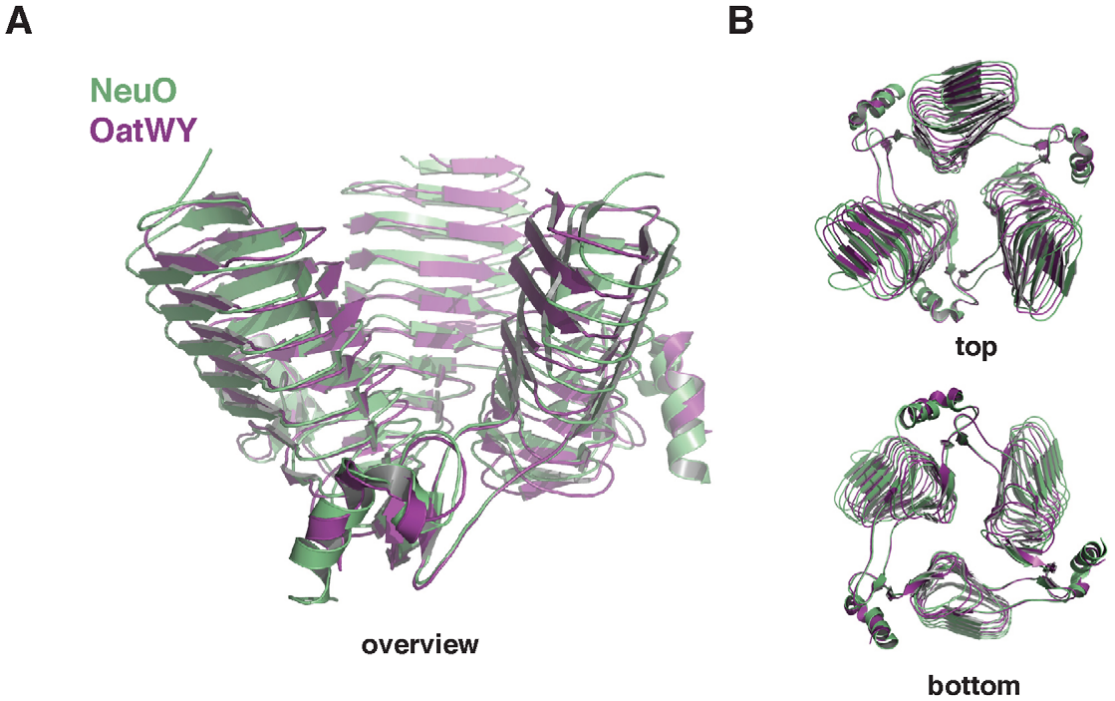
Structural comparison between NeuO and OatWY. A least-squares superimposition of NeuO (green) (PDB-ID: 3JQY) and OatWY (magenta) (PDB-ID: 2WLD) reveals the high overall similarity between both molecules. Irrespective of their different substrate specificities the monomers of both proteins can be superimposed with an r.m.s.d. of 0.9 Å. Globally both proteins share a high inclination between the chains, probably as an adaptation to their unusually long substrates. The least-squares superimposition was calculated using the program LSQMAN [45].

Comparison with other members of the LβH-family revealed that the sialate O-acetyltransferase OatWY of serogroup W-135 and Y meningococci [25] is the closest structural homologue of NeuO. The individual monomers can be superimposed with a root-mean-square distance (r.m.s.d) of 0.91080 Å (**Figure 4**). Moreover, the quaternary structure of both enzymes is characterized by a tilted arrangement of the subunits with an inclination between the long axes of the monomers of 45° and 34° in NeuO and OatWY, respectively.

Within the active sites of NeuO and OatWY, six amino acid residues are highly conserved (see boxed residues in **Figures 3A and 3B**). In addition to the catalytically important residues His147 (His121 in OatWY) and Trp171 (Trp145 in OatWY), these include Met134 (Met108 in OatWY), Arg174 (Arg148 in OatWY), Trp220 (Trp195 in OatWY), and Arg222 (Arg197 in OatWY). In both enzymes, Trp220/195 forms a hydrophobic pocket in front of the active site which could be important for trapping the sialic acid moiety currently positioned in the active site in the right orientation. Such a binding mode is found in sialidases which posses a hydrophobic pocket in the active site to accommodate the N-acetyl group of sialic acid [26], [27]. Met134/108 located next to Trp220/195 could contribute to the hydrophobic pocket but in addition, this residue has a prominent position within the catalytic center of both NeuO and OatWY, pointing towards the active site residues His147/121 and Trp171/145.

Arg222/197 positioned in the loop between α1 and β25 (residues 221–232 in NeuO) forms a polar contact with Thr113/87 located in the LβH-domain of the same subunit, thereby stabilizing the orientation of this loop. Moreover, the main chain carbonyl oxygen of Arg222/197 is in hydrogen bond distance (2.84 Å) to the catalytic residue His147/121 from the adjacent subunit and might be relevant for the proper positioning of the catalytic histidine.

As mentioned above, Arg174 forms an intersubunit salt bridge with Glu119 at one out of three interfaces in NeuO. In OatWY, however, this salt bridge is found symmetrically at all three interfaces and is formed by the corresponding residues Arg148 and Glu92. This salt bridge could be relevant for the correct quaternary structure of both enzymes and might be broken up at two interfaces of NeuO only due to the crystalline environment.

Outside of the catalytic center, only one of the positively charged residues forming the proposed polySia binding site of NeuO is conserved in OatWY: Arg111 at the entrance of the positively charged platform, which corresponds to Lys85 in OatWY. This might reflect the differences in acceptor substrate specificities of the two enzymes.

## Discussion

**Figure 5:**
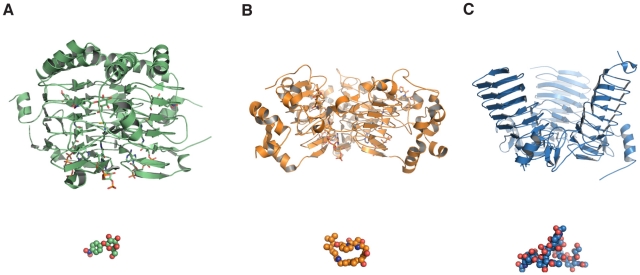
Inclination angles of LβH-acetyltransferases correlate with the size of the acceptor substrate. (A) The galactoside acetyltransferase GAT (PDB-ID: 1KRV) [23]. (B) The streptogramin acetyltransferase Vat(D) (PDB-ID: 1KHR) [24]. (C) The polysialic acid acetyltransferase NeuO (PDB-ID: 3JQY). The different structures are displayed in cartoon mode with their individual substrate displayed as spheres in the same color. Whereas the LβH-fold is highly conserved also with respect to the acetyl-CoA binding site, the inclination angle between the subunits increases with the size of the acceptor substrate and is by far most pronounced in NeuO.

In this study, we have solved the structure of the prophage-encoded O-acetyltransferase NeuO responsible for phase-variable capsule modification of the neuroinvasive pathogen *E. coli* K1. The homotrimeric enzyme shares typical features of the LβH-family of acyltransferases combined with an unusual funnel-shaped outline. To highlight structural characteristics of polySia-specific LβH-acetyltransferases, the structure of NeuO was compared with other members of the LβH-family. The closest structural homologue of NeuO was found to be the recently crystallized sialate O-acetyltransferase OatWY of *N. meningitidis* serogroup W-135 and Y [25]. OatWY is specific for the glucose and galactose containing sialic acid heteropolymers [-6-Glc-α1,4-Neu5Ac-α2-]_n_ and [-6-Gal-α1,4-Neu5Ac-α2-}_n_], respectively, and catalyzes the transfer of O-acetyl groups from acetyl-CoA to O7 and O9 of the sialic acid moieties. Notably, both NeuO and OatWY differ markedly from other members of the LβH-family with respect to the geometry of the LβH-domain and the orientation of the three subunits. Whereas the common LβH-fold has an equilateral, triangular cross-section [18], this geometry is distorted in NeuO and OatWY and a bean shaped cross-section is observed. Moreover, the parallel arrangement of the individual monomers is lost and the monomers are arranged with a notable inclination of 45° and 34° between each chain in NeuO and OatWY, respectively [25]. Although the acceptor substrates of both enzymes differ in composition and glycosidic linkage of the Neu5Ac residues, both are large polymers. Comparison of LβH-acetyltransferases with regard to their acceptor substrates suggests a correlation between tilted subunit arrangement and substrate size. While the subunits of acetyltransferases with small substrates, like the galactoside acetyltransferase GAT of *E. coli* are oriented in parallel to each other [28], a more opened subunit orientation is observed in enzymes with bulkier substrates such as the streptogramin acetyltransferase Vat(D) of *Enterococcus faecium* (**Figure 5**) [29]. However, the by far most pronounced inclination between the subunits is found in NeuO and OatWY, apparently in order to accommodate their particularly long polysaccharide substrates.

Interestingly, both NeuO and OatWY share a methionine residue (Met134 and Met108 in NeuO and OatWY, respectively) which occupies a prominent position in the catalytic center. Examination of related structures reveals that a similarly positioned methionine is only found in LβH acetyltransferases that act on di- or oligosaccharides, such as the maltose O-acetyltransferase of *E. coli* (MAT - Met100) and the galactoside acetyltransferase of *E. coli* (GAT – Met127) [28]. For none of these enzymes structures containing the respective natural acceptor oligosaccharide are available. However, in the structure of GAT in complex with the non-physiological acceptor substrate *p*-nitrophenyl-β-D-galactopyranoside (PNPβGal) the sulfur of Met127 points towards the oxygen forming the ether bond between PNP and galactose (PDB-ID: 1KVR), suggesting that a non-canonical type of electrostatic interaction may coordinate this bond. Non-bonded interactions between a divalent sulfur (S) and an oxygen (O) atom have been described previously [30], [31] and a respective analysis carried out for protein-ligand complexes derived from the crystal structures in the Protein Data Bank revealed many cases of non-canonical S-O interactions involving the sulfur atom of methionine [32]. Most prominent were interactions with an ether oxygen atom in the ligand with a stereochemistry remarkably similar to the one observed for the Met127 ether oxygen interaction observed in the PNPβGal-GAT complex. This opens the possibility that in acetyltransferases acting on di- and oligosaccharides, the structurally conserved methionine may coordinate the glycosidic bond and thereby fulfill an important role in proper positioning of the acceptor sugar within the active site.

**Figure 6:**
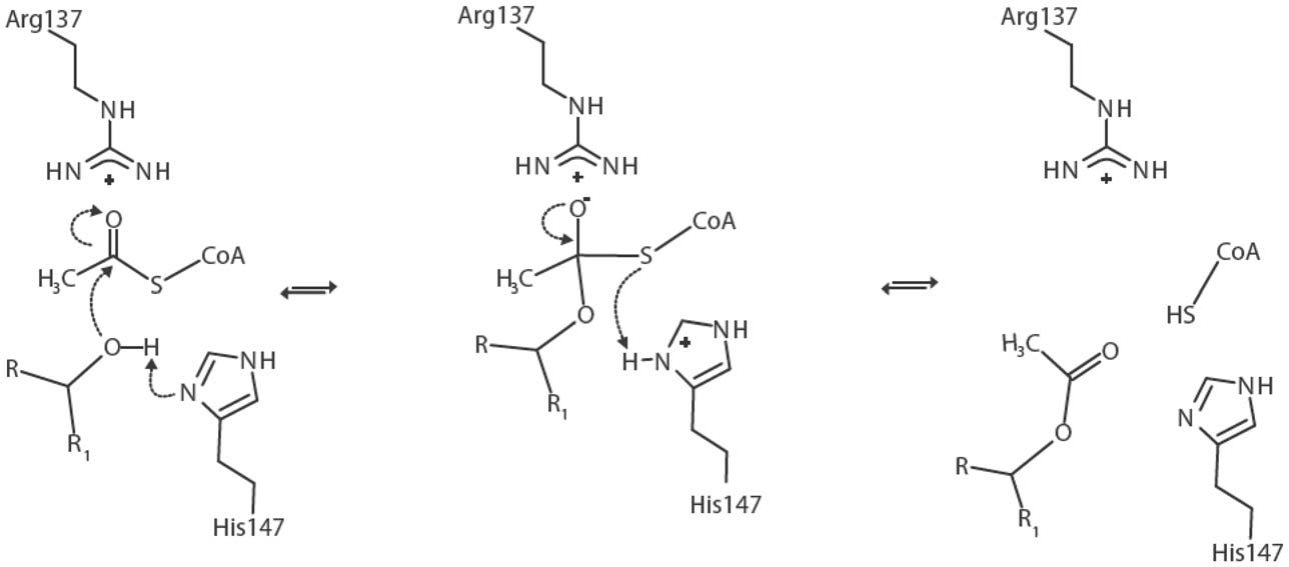
Proposed reaction mechanism for polySia O-acetylation catalyzed by NeuO. Activation of the hydroxyl group at position C7 or C9 of the sialic acid moiety currently positioned in the active site of NeuO facilitates nucleophilic attack of the thioester carbonyl of acetyl-CoA. This leads to the formation of a sialyl-CoA intermediate with Arg137 stabilizing the oxyanion. Decomposition of the tetrahedral intermediate results in release of free CoA and O-acetylated polySia. During this step, His147 protonates the sulfur atom of CoA and a new cycle of acetylation can begin.

So far, all members of the LβH family that have been crystallized share a catalytic histidine positioned in a loop protruding out of the LβH domain. Moreover, family members that use acetyl-CoA as donor substrate contain, in addition, a conserved tryptophan which is located opposite to the catalytic histidine but is contributed by the adjacent subunit [28], [33]–[35]. Previously, it was shown that His147 and Trp171 play a crucial role for NeuO activity [14]. The structural data show that both residues are located in the proposed active site in positions highly conserved among acetyltransferases of the LβH family (MAT [35], GAT [28], VatD [29], PaXAT [33], and OatWY [25]). On the basis of the structural data, the following catalytic mechanism can be proposed for NeuO (**Figure 6**): (i) after binding of both substrates, His147 activates the hydroxyl group at position C-7 or C-9 of the Neu5Ac moiety currently positioned in the active site. This increases the nucleophilicity of the oxygen atom, thereby facilitating the attack of the thioester carbonyl of acetyl-CoA, (ii) resulting in the formation of a sialyl-acetyl-CoA intermediate. In this intermediate the thioester carbonyl adopts a tetragonal conformation with the carbonyl oxygen present as an oxyanion. (iii) In the final step of the reaction, His147 protonates the sulfur atom of CoA, releasing both substrates from the active site. Since the nucleophilic attack changes the hybridization state of the acetyl carbon from trigonal to tetragonal, it is difficult to unambiguously determine the residues that stabilize the intermediate oxyanion. It is conceivable that either the N_ε_ of Arg137 or Arg174 or the positive charge of Arg137 directly stabilizes the sialyl-CoA intermediate. A similar reaction mechanisms has been suggested for the serine acetyltransferases SAT of *Haemophilus influenzae*, and in this case, stabilization of the oxyanion by backbone amides was discussed [36].

Within the LβH-family, NeuO stands out with respect to its unique regulatory mechanism based on the variable N-terminal poly-ψ-domain formed by tandem copies of an RLKTQDS heptad. Due to slipped strand mispairing within the 5′-VNTR region that encodes the poly-ψ-domain, a multitude of NeuO variants can be generated and so far, heptad numbers between 1 and 31 (corresponding to 3 to 93 nucleotide repeats) were observed in naturally occurring isolates [13]. Although the presence of the poly-ψ-domain is not a prerequisite for NeuO activity, the catalytic efficiency increases linearly with the number of heptads [14]. To explain this effect, previous studies proposed a model in which the poly-ψ-domains of a functional trimer assemble into a triple coiled-coil [9], [14]. However, the structural data do not confirm this model. Albeit the structure of the first 18 residues could not be solved for the apo-enzyme, the overall outline of NeuO with the N-termini of the three subunits pointing almost 65 Å away from each other, exclude the formation of a triple coiled-coil. Assuming the proposed extended mainly positively charged polySia binding site which involves N-terminally located residues such as Arg73 and Lys95, the N-terminal poly-ψ-domain might contribute to polySia binding by forming an extension of the acceptor binding site. Notably, each heptad contains two positively charged amino acids (Arg in position 1 and Lys in position 3 of the heptad), which could mediate interaction with the negatively charged carbohydrate polymer. *In vitro*, NeuO acts exclusively on sialic acid oligomers containing ≥14 residues [10], [14], indicating that O-acetylation *in vivo* is a co- or post-synthetic process. The transfer of multiple acetyl groups per chain can be catalyzed either (i) by a processive mode with the growing polymer (which is elongated on the non-reducing end) gliding through the enzyme from the N-terminal binding site via the active site towards the positively charged platform or (ii) distributively by release of the polymer after each O-acetylation step followed by a new round of attachment and transfer. The flexible N-terminal poly-ψ-domain might facilitate the disentangling of the incoming polySia chains, gliding along the polymer and/or attachment to a new polymer chain, and thereby regulate NeuO activity.

## Materials and Methods

### Generation of constructs for phase-stable expression of NeuO

All constructs used in this study were based on pET22Δ, a modified pET22b vector (Novagen) in which the pelB leader sequence was removed by ligation of the oligonucleotides AKB42/AKB43 into the XbaI/BamHI sites of pET22b. pET22Δ-NeuO+0 carrying *neuO* without 5′-VNTR-region was generated by subcloning of the BamHI/XhoI fragment of pET22Strep-NeuO+0 [14] into the corresponding sites of pET22Δ resulting in a plasmid which allows the expression of NeuO with a C-terminal hexahistidine tag. For phase-stable expression of the variant NeuO+12 comprising four N-terminal RLKTQDS heptads, the 12 tandem copies of the naturally occurring heptanucleotide sequence 5′-AAGACTC-3′ was replaced by an artificial sequence that still encoded four RLKTQDS heptads but lacked repetitive DNA elements. In the first step, the oligonucleotides AKB146/AKB147 encoding one heptad and comprising an AflII site were inserted into the BamHI site of pET22Δ-NeuO+0. The introduced second BamHI site was eliminated by fusion PCR using the primer pairs T7/AKB149 and pET-RP/AKB148. Purified PCR-products were joined by PCR and the fragment was amplified by nested PCR with the primers T7 and AKB3. The resulting PCR-product was subcloned into the BamHI/XhoI sites of pET22Δ. In a second step, the oligonucleotides AKB152/AKB153 encoding three RLKTQDS heptads were inserted by adapter ligation into the BamHI/AflII sites resulting in the plasmid pNeuO+12s. The identity of this construct was confirmed by sequencing. Sequences of all oligonucleotides are given in Table S1.

### Expression and purification of NeuO+12

*E. coli* BL21 gold (DE3) (Novagen) transformed with pNeuO+12s were grown at 37°C in 2 L of Power Broth (AthenaES) containing 100 µg/ml carbenicillin. At an optical density (OD_600_) of 1.8, expression was induced by adding 0.1 mM IPTG and bacteria were cultivated at 15°C for 20 h. Bacteria were harvested by centrifugation, lysed by sonication, and the soluble fraction was applied to a 5 ml HisTrap-chelating HP column (GE Healthcare). After washing with 25 ml of binding buffer (20 mM Tris-HCl pH 7.5, 500 mM NaCl, 40 mM imidazole), proteins were eluted with a linear imidazole gradient (40 to 500 mM imidazole in binding buffer). Enzyme-containing fractions were pooled and loaded immediately onto a HiLoad™ 16/60 Superdex™ 200 size-exclusion chromatography column (GE Healthcare) equilibrated with 10 mM Tris-HCl pH 7.5 and 150 mM NaCl at 4°C. NeuO-containing fractions were pooled, concentrated to 6.3 mg/ml by ultra filtration (Amicon Ultra-15 ultracel-10k, Millipore), aliquoted, and subsequently flash-frozen in liquid nitrogen.

### Crystallization and data collection

Crystallization was performed at 20°C in sitting drop vapour diffusion plates. The protein was concentrated to a final concentration of 6.3 mg/ml and mixed with equal volumes of precipitant solution (0.1 M Tris pH 7.2; 0.45 M Glycine; 0.2 M NH_4_NO_3_; 7% PEG4000 (w/v)). Crystals suitable for data collection experiments appeared randomly and scarcely after 1 week and grew to a final size of approximately 150 µm×50 µm×75 µm. Crystals were cryo-protected by soaking in a solution of mother-liquor containing 20% glycerol. Cryo-protected crystals were flash frozen in liquid nitrogen prior to data collection. Data collection was performed at 100 K at beamline 14.1 at Bessy Berlin.

### Data processing, phasing and density modification

X-ray diffraction data were indexed and integrated in the XDS suite [37], [38] and scaled in ScalA [39]. Phases were obtained by molecular replacement in MOLREP [40] using a search model generated by homology modeling [41]. Initially a solution was found in a crystal that crystallized in space group P6_3_ (data not shown). The partial model derived from this solution was subsequently used to phase the highest resolution datasets of the crystal form P2_1_. Model building was performed in Coot [42], density modification was performed in Parrot [39], refinement was performed in Phenix [43], and stereochemistry was analyzed in Rampage [39]. Molecular Images were generated in Pymol (DeLano Scientific).

### PDB Accession code

Atomic coordinates and structure factors were deposited at the Research Collaboratory for Structural Bioinformatics (RCSB) Protein Databank (PDB) and are available under the accession number 3JQY.

## Supporting Information

Table S1 Sequences of sense (s) and antisense (as) oligonucleotides.
(DOCX)

Figure S1 **Stereochemistry Analysis of NeuO.** All residues could be refined to good stereochemistry - no residues can be found in the disallowed region.
(TIFF)

Figure S2 **N-terminal flexibility of NeuO.** The 18 N-terminal amino acids are missing in the crystal structure of NeuO indicating an intrinsic flexibility of the poly-ψ-domain. The partial ordering of residues 19–29 of chain A, visible as strands β1 and β2, is likely to be an effect of crystal packing.
(TIFF)
