# Supplementary material for: Crystal Structure Analysis of the Polysialic Acid Specific O-Acetyltransferase NeuO
Source: PLoS One. 2011 Mar 1;6(3):e17403. doi: 10.1371/journal.pone.0017403 (PMC3046976; doi:10.1371/journal.pone.0017403)
Supplement: Table S1 — Sequences of sense (s) and antisense (as) oligonucleotides. (DOCX) [file pone.0017403.s001.docx]

**Table S1.** Sequences of sense (s) and antisense (as) oligonucleotides.

| Name |  | Sequence |
| --- | --- | --- |
| AKB 42 | s | 5’-CTAGAAATAATTTTTCGCGACTTTAAGAAGGAGACG-3’ |
| AKB 43 | as | 5’-GATCCGTCTCCTTCTTAAAGTCGCGAAAAATTATTT-3’ |
| AKB 146 | s | 5’-GATCCATGCTGCGTCTTAAGACCCAGGATTCGG-3’ |
| AKB 147 | as | 5’-GATCCCGAATCCTGGGTCTTAAGACGCAGCATG-3’ |
| AKB 148 | s | 5’-TAAGACCCAGGATTCGttttccgttgatgataatggg-3’ |
| AKB 149 | as | 5’-tatcatcaacggaaaacgaatcctgggtcttaagac-3’ |
| AKB 152 | s | 5’-GATCCATGCTGCGTCTCAAGACACAAGACAGCAGGCTAAAGAC GCAGGATTCTCGATTGAAAACTCAAGACAGTCGCC-3’ |
| AKB 153 | as | 5’-TTAAGGCGACTGTCTTGAGTTTTCAATCGAGAATCCTGCGTCT TTAGCCTGCTGTCTTGTGTCTTGAGACGCAGCATG-3’ |
| T7 | s | 5’-taatacgactcactataggg-3’ |
| pET-RP | as | 5’-CTAGTTATTGCTCAGCGG-3’ |
| AKB 219 | as | 5’-CCAATAGGCCGAAATCGGC-3’ |
| AKB 217 | s | 5’-GCAAGGGATCCATGCTGCGTCTC-3’ |
| AKB 218 | as | 5’-CGGTCCTCGAGTTGCGTGAGCTTC-3’ |
