# Supplementary figures and images for: Crystal Structure Analysis of the Polysialic Acid Specific O-Acetyltransferase NeuO
Source: PLoS One. 2011 Mar 1;6(3):e17403. doi: 10.1371/journal.pone.0017403 (PMC3046976; doi:10.1371/journal.pone.0017403)

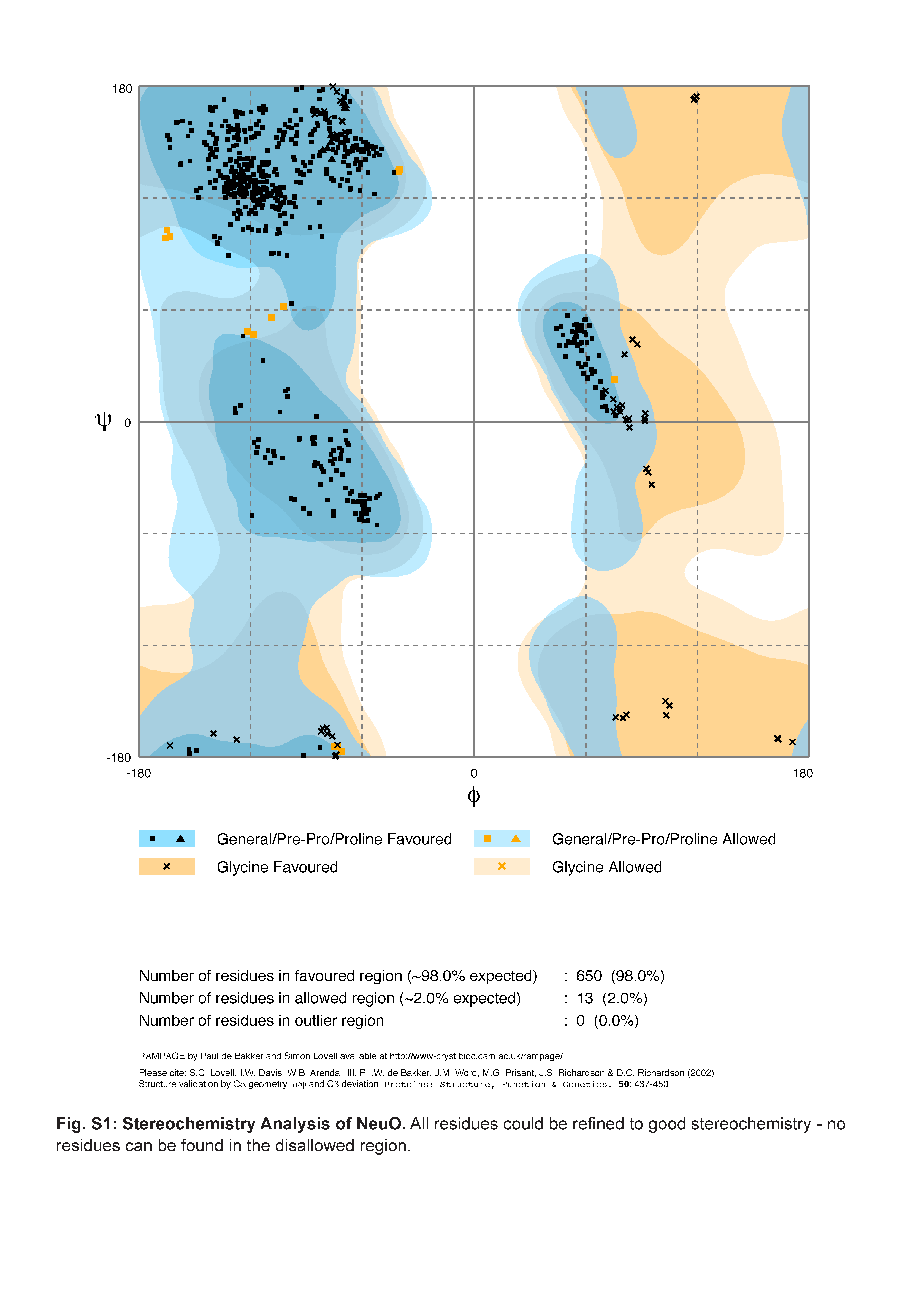

Supplement: Figure S1 — Stereochemistry Analysis of NeuO. All residues could be refined to good stereochemistry - no residues can be found in the disallowed region. (TIFF) [file pone.0017403.s002.tiff]

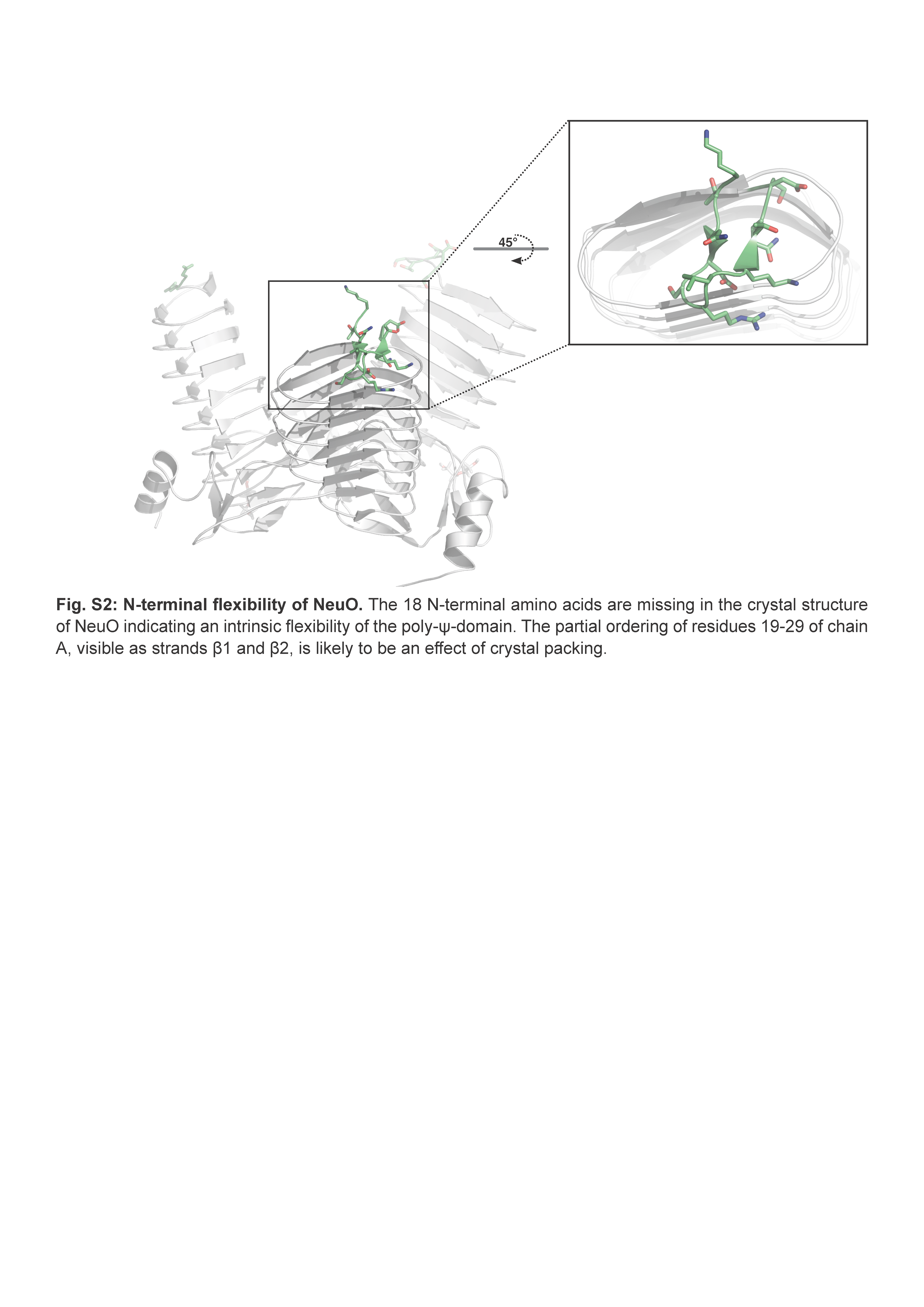

Supplement: Figure S2 — N-terminal flexibility of NeuO. The 18 N-terminal amino acids are missing in the crystal structure of NeuO indicating an intrinsic flexibility of the poly-ψ-domain. The partial ordering of residues 19–29 of chain A, visible as strands β1 and β2, is likely to be an effect of crystal packing. (TIFF) [file pone.0017403.s003.tiff]
